# Integrated Diagnostic Approach Using Basophil Activation Test and IgE Assays for Shrimp and Prawn Allergy †

**DOI:** 10.3390/medicina61061040

**Published:** 2025-06-05

**Authors:** Nhu N. Q. Nguyen, Thao H. Nguyen, Minh K. Le, Tram B. Duong, Duy L. Pham, Tai T. Tran, Tu H. K. Trinh

**Affiliations:** 1 Center for Molecular Biomedicine, University of Medicine and Pharmacy at Ho Chi Minh City, Ho Chi Minh City 70000, Vietnam; nnqnhu@ump.edu.vn (N.N.Q.N.); lkminh@ump.edu.vn (M.K.L.); dbtram@ump.edu.vn (T.B.D.); 2 Department of Physiology-Pathophysiology-Immunology, Faculty of Medicine, School of Medicine and Pharmacy, Tra Vinh University, Tra Vinh 87000, Vietnam; nhthao@tvu.edu.vn; 3 Faculty of Medicine, University of Medicine and Pharmacy at Ho Chi Minh City, Ho Chi Minh City 70000, Vietnam; 4 University Medical Center Ho Chi Minh City, Ho Chi Minh City 70000, Vietnam; tai.tt1@umc.edu.vn

**Keywords:** shellfish allergy, basophil activation test, food allergy, Vietnam

## Abstract

*Background and Objectives*: Shellfish allergies are common in Vietnam. The basophil activation test (BAT) is a powerful tool in the diagnosis of food allergies. We aimed to evaluate the application of BAT to distinguish shrimp allergy in comparison with skin prick test and specific IgE measurement. *Materials and Methods*: We recruited adult shrimp- or prawn-allergic subjects from the University Medical Center (Vietnam). BAT was performed using the in-house crude extracts for two allergens: black tiger shrimp *Penaeus monodon* (shrimp) and giant freshwater prawn *Macrobrachium rosenbergii* (prawn). The percentages of CD63 in response to shrimp and prawn were recorded. The results of skin prick tests (SPT) and the specific IgE (sIgE) levels in response to commercial shrimp/prawn were noted. Receiver operating characteristic (ROC) analysis and area under the curve (AUC) were calculated. *Results*: Of 43 recruited subjects, 9 (26.5%) subjects had a specific allergy to shrimp, 2 (5.9%) subjects had a specific allergy to prawn, and 23 (67.6%) subjects had both shrimp and prawn allergy. Basophil CD63% was significantly increased in subjects with allergy to shrimp and prawn (*p* < 0.05% for all). Compared with SPT and sIgE, CD63 expression-based BAT was better in discriminating subjects with allergies to these species from their non-allergic counterparts (AUC/sensitivity/specificity = 0.88/77%/89% for shrimp, and 0.74/88%/77% for prawn, *p* < 0.05 for all). The addition of SPT and BAT improved the diagnostic power. A positive BAT could help identify shrimp/prawn allergy among cases with negative SPT/sIgE to shrimp/prawn. BAT facilitated the diagnosis of shrimp allergy among prawn-allergic subjects (100% accurate). *Conclusions*: The BAT test can help predict clinical reactions to shrimp and prawn in allergic patients, and enhance diagnostic accuracy in cases where SPT or specific IgE tests yield negative results.

## 1. Introduction

Seafood allergy is a prevalent and potentially life-threatening condition with an increasing global prevalence [1]. Clinical symptoms ranging in intensity from moderate to life-threatening, such as gastrointestinal problems, urticaria, and airway inflammation, can be triggered by allergenic foods [2]. The prevalence of food allergies is increasing among both adults and children. The World Allergy Organization states that approximately 550 million people worldwide are affected by food allergies, and approximately 2.5% of the global population experience adverse reactions to seafood [3,4]. The incidence of seafood allergies in the United State is higher in adults compared to children (2.5% vs. 1.3%), [4,5]. Several studies have been conducted on the epidemiology and clinical characteristics of seafood allergies in different countries, with some fish and seafood allergens unique to those regions being characterized [6]. Furthermore, seafood allergy is predominant in Asian populations, with shellfish being the region’s most common food allergen [7,8]. Thus, the accurate diagnosis of seafood allergies is crucial for the effective management and avoidance of allergic reactions.

Traditional diagnostic methods, such as skin prick tests (SPT) and specific IgE quantification, have limitations in diagnosing seafood allergies due to their relatively low specificity [9,10,11]. In this context, the basophil activation test (BAT) has emerged as a promising tool for diagnosing seafood allergy [12]. BAT is a flow-cytometry-based functional assay that assesses the degree of basophil activation upon exposure to specific allergens [12]. The test has demonstrated high reproducibility and feasibility in clinical settings [13].

The use of BAT in diagnosing seafood allergy is supported by its ability to reproduce IgE-mediated allergic reactions in vitro, providing clinically relevant insights into patient reactivity [14]. Additionally, BAT has been utilized to diagnose allergies to other food types, such as eggs and peanuts, highlighting its versatility in food allergy diagnosis [15]. The ability of the test to identify patients with active autoimmune chronic spontaneous urticaria further underscores its clinical utility in allergic disease diagnosis [16].

Although there have been many studies on food allergies worldwide, research in Vietnam remains limited. Seafood sources in Vietnam are diverse, and not all allergens are included in the commercial kits. This has led to difficulties for clinicians approaching seafood-allergic patients in Vietnam. While oral food challenge (OFC) remains the gold standard, it is time-consuming and easily rejected by patients. Therefore, the role of IgE assays becomes crucial prior to conducting OFC. However, the allergen compositions in commercial extracts are highly variable and may be insufficient for diagnosis [17,18]. Using the fresh food or in-house allergens representing regional diversity may improve diagnostic capacity [19,20]. We established the in-house allergens, which have been used for SPT and BAT. Among the food allergens, previous work demonstrated that shrimps were common among Vietnamese food-allergic subjects. The black tiger shrimp (shrimp) (*Penaeus monodon*) and giant freshwater prawn (prawn) (*Macrobrachium rosenbergii*) are frequently reported on in the Vietnamese seafood-allergic population [21]. This study was performed to evaluate the use of BAT, SPT and sIgE assays in discriminating shrimp- and prawn-allergic subjects.

## 2. Materials and Methods

### 2.1. Subject Recruitment and Allergic Testing

We recruited patients with a history of shrimp or prawn allergies from the Unit of Allergy and Clinical Immunology, University Medical Center (Vietnam), within the 2-year period 2021–2022. The patients were aged between 18 and 60 years old. The patients reported recurring (at least 3 times) events of acute onset symptoms within minutes to 2 h of exposure to the same species, either shrimp or prawn. Except for cases with a history of anaphylaxis or severe allergic reactions in the past 6 months [22], patients were exposed to cooked shrimp or prawn (weighed approximately 15 g), divided into four incremental doses administered every 15 min under an investigator’s supervision. Two challenges with each species were conducted 2–4 weeks apart. Those who had either anaphylaxis/severe allergic reactions or developed reactions (presence of objective singes or persistent/worsening subjective symptoms according to PRACTALL consensus [23,24]) after consumption were defined as allergic. Conversely, those who did not meet the above criteria were considered non-allergic. All patients were given informed consent prior to participating in the study. Data on demographic characteristics and history of seafood allergies were collected. SPTs were performed according to the European Academy of Allergy and Clinical Immunology (EAACI) in the University Medical Center, Ho Chi Minh City, Vietnam [25]. The tested allergens included *Dermatophagoides pteronyssinus*, *Dermatophagoides farinae*, and the in-house crude extracts of shrimp and prawn. The in-house crude extracts were prepared as previously described [26,27].

Blood sampling was performed for the measurement of total IgE, sIgE to shrimp, and BAT. The above tests were conducted at the Center for Molecular Biomedicine (University of Medicine and Pharmacy at Ho Chi Minh City, Ho Chi Minh City, Vietnam). Briefly, 5 mL of the patient’s whole blood was collected in blood collection tubes without EDTA. Subsequently, the collected samples were left undisturbed for 15–30 min at room temperature and centrifuged at 3000 rpm for 10 min in a refrigerated centrifuge. The layers containing buffy coat and erythrocytes were processed for BAT. The obtained plasma was used to measure total IgE and specific IgE concentrations (EUROIMMUN, Lubeck, Germany) following the manufacturer’s instructions, as described in more detail below.

This study was approved by the Ethics Committee of University of Medicine and Pharmacy at Ho Chi Minh City, Vietnam (57/HĐĐĐ-ĐHYD, 17 January 2022).

### 2.2. Measurement of Total IgE and sIgE

The plasma sample was analyzed for specific IgE concentration (sIgE) using the immunoblot method with the EUROLINE Atopy Food “South East Asia 1” IgE kit (DP 3411-1601 E, EUROIMMUN, Germany) according to the manufacturer’s instruction. Among the 20 allergens in the panel, we chose the sIgE to shrimp/prawn to analyze for this study. The concentrations (IU/mL) were measured using undiluted serum, and the sIgE > 0.35 IU/mL was considered positive. The cross-reactive carbohydrate determinant (CCD) markers were included in the specific IgE assays for all samples to assess potential CCD reactivity. None of the samples tested positive for CCD, and therefore CCD inhibition was not required and not performed.

Regarding total IgE, the plasma sample was analyzed by ELISA using the total IgE kit (EV 3840-9601 E, EUROIMMUN, Lübeck, Germany) following the manufacturer’s instructions. Briefly, the kit contained tagged capture antibodies binding to the target proteins and detection antibodies conjugated with dyes for specific immune reaction. The complete immune complex was immobilized via anti-tag antibodies coated at the bottoms of the wells. Samples (dilute 1:10 in sample buffer) and standards (100 μL/well) were added and incubated for 30 min at room temperature. For samples that exceeded the upper detection limit of total IgE following an initial 1:10 dilution, additional dilutions were performed up to 1:30 to ensure accurate quantification. Each sample was measured in two replicates, and the average value was used for analysis. The well plate was washed and followed by an incubation of 100 μL enzyme conjugate (peroxidase-labeled anti-human IgE) for another 30 min. After incubation, the wells were washed to remove unbound substances. A tetramethylbenzidine (TMB) development solution (100 μL/well) was then added as a subsequent incubation step, which was catalyzed by hydrogen peroxide to produce a blue color. The reaction was terminated by adding a stop solution (100 μL/well), changing the color from blue to yellow. The OD signal, measured at a wavelength of 450 nm, was directly proportional to the amount of total IgE bound.

### 2.3. Basophil Activation Test

Seafood allergens were purified following the conventional protocol of our laboratory at the Center for Molecular Biomedicine, University of Medicine and Pharmacy, Ho Chi Minh City. SDS-PAGE of the in-house crude extracts were included in the Supplementary Materials. BAT was performed as described in our previous study [27]. Briefly, the buffy coats were collected from 5 mL peripheral blood of each patient by hypotonic lysis with distilled water. The leukocytes then were primed with interleukin (IL)-3 (2 ng/mL) (Invitrogen, Thermo Fisher Scientific, Waltham, MA, USA) for 10 min at 37 °C. Subsequently, the leukocytes were incubated with different seafood extracts (50 µg/mL) for 30 min at 37 °C. Anti-IgE antibody (50 µg/mL) served as a positive control and without any allergen as a negative control. Fluorescein isothiocyanate (FITC)-conjugated anti-CD123 (Invitrogen), allophycocyanine (APC)-conjugated anti-HLA-DR (Invitrogen), peridinin chlorophyll protein-cyanine5.5 (PerCP-Cy5.5)-conjugated anti-CD63 (Invitrogen) and phycoerythrin (PE)-conjugated anti-CD203c (Invitrogen) antibodies were added for incubation during the reaction. The samples were then analyzed by flow cytometry (BD FACSCanto II Flow cytometer, Becton, Dickinson and Company—BD Biosciences, Franklin Lakes, NJ, USA). Basophils were identified based on the forward and side scatter CD123^pos^/HLA-DR ^neg^. Basophil activation was determined as the percentages of activated basophils markers CD63 (CD63%) and CD203c (CD203c%). Other reagents were from Merck KGaA (Darmstadt, Germany) unless otherwise indicated.

To exclude low responders, the fluorescence of basophils in response to anti-human IgE antibody was divided into the fluorescence of unstimulated basophils. If the ratio was <10%, subjects were defined as low responders and excluded from the study [28].

### 2.4. Statistical Analysis

Data analysis was performed using JASP Team (2024), JASP (Version 0.18.3) (computer software) [29]. Quantitative variables were displayed as the median and standard error, and categorical variables were presented as counts and percentages (%). The Shapiro–Wilk test was employed to assess the normality of the data. For parametric analysis, the Student’s *t*-test was utilized, whereas the Mann–Whitney U test was applied for non-parametric analysis. For categorical variables, comparisons between groups were calculated using Pearson’s chi-squared tests. Receiver operating characteristic (ROC) curves were used to determine the diagnostic values of SPT, specific IgE levels, and BAT. The optimal cut-off points for each test were chosen based on Youden’s J statistic, computed as sensitivity + specificity − 1. The graphs were prepared by GraphPad Prism 6.05 for Windows (GraphPad Software Inc., Boston, MA, USA) Statistical significance was set at *p* < 0.05.

## 3. Results

### 3.1. Demographic Characteristics and Classifications of Participants

Forty-three subjects were included in this study, and the proportions of allergies to shrimp and prawn were 74.41% and 58.14%, respectively. There were 9 (26.5%) subjects who had a specific allergy to shrimp, 2 (5.9%) subjects had a specific allergy to prawn, and 23 (67.6%) subjects had both shrimp and prawn allergies (Table 1).

The median age of the patients was 29 ± 8.66 years, and female patients were predominant (67.44%). The proportion of comorbid fish allergy was 6/43 (6.98%), while none of the subjects reported a mollusk allergy. The median total IgE level was 1222 ± 3216.82 IU/mL.

### 3.2. SPT, sIgE and BAT Indices in Shellfish Allergy Versus Non-Allergy

**Figure 1:**
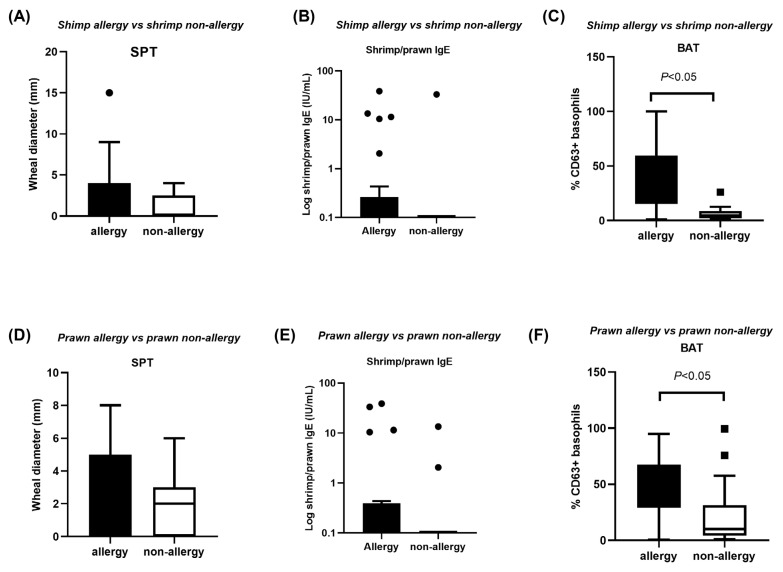
Indices of SPT, sIgE to shrimp/prawn, and BAT. (**A**–**C**) Comparison between shrimp-allergic and non-allergic subjects; (**D**–**F**) comparison between prawn-allergic and non-allergic subjects. *p*-values were calculated by Mann–Whitney U test between groups. SPT and BAT results are presented by the wheal diameter (mm) and %CD63 expression on basophils, respectively. Serum shrimp/prawn IgE values are shown as log-transformed data. BAT, basophil activation test; prawn, giant tiger prawn; shrimp, black tiger shrimp; SPT, skin prick test.

In all cases, SPT tended to be higher in shrimp-allergic vs. shrimp-non-allergic subjects (3 ± 0.58 vs. 0.00 ± 0.80, *p* = 0.086) (Figure 1A), and in prawn-allergic vs. prawn-non-allergic subjects (3 ± 0.55 vs. 2 ± 0.49, *p* = 0.33) (Figure 1D). There were no significant differences in terms of sIgE to shrimp/prawn between shrimp-allergic and shrimp-non-allergic subjects (0.00 ± 7.45 vs. 0.00 ± 10.03, respectively) (*p* = 0.38) (Figure 1B), and between prawn-allergic and prawn-non-allergic subjects (0 ± 10.14 vs. 0.00 ± 3.17, respectively) (*p* = 0.21) (Figure 1E).

The percentage of CD63 (%CD63) was noted. The %CD63 expression on basophils was enhanced significantly in shrimp- or prawn-allergic subjects vs. non-allergic counterparts (*p* = 0.003, *p* = 0.04, respectively). Specifically, the levels of %CD63 on basophils from shrimp-allergic subjects were 44.80 ± 5.16, and on those from shrimp-non-allergic subjects they were 6 ± 3.18. Regarding the BAT to prawn, the levels of %CD63 on basophils from prawn-allergic subjects were 40.10 ± 5.51, and on those from prawn-non-allergic subjects they were 12.40 ± 8.23. (Figure 1C,F).

### 3.3. Diagnostic Values of BAT in Discriminating Shellfish-Allergic from Non-Allergic Subjects

**Figure 2:**
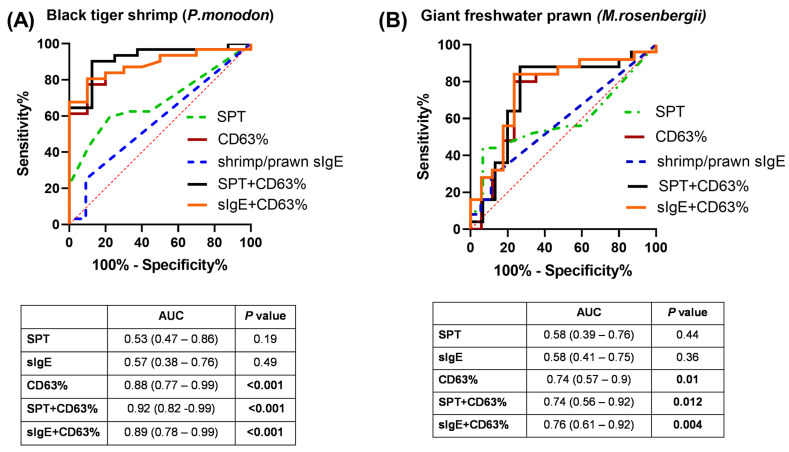
ROC curve analysis of SPT, BAT (%CD63), shrimp/prawn sIgE and the combination between SPT/sIgE and BAT (%CD63) in discriminating either (**A**) shrimp-allergic or (**B**) prawn-allergic subjects from their non-allergic counterparts. AUC, area under the curve.

The diagnostic performance of CD63% on basophils in comparison to SPT and shrimp/prawn sIgE was analyzed using the ROC curve to discriminate shrimp- and prawn-allergic subjects from non-allergic subjects (Figure 2 and Table 2). In Figure 2, the CD63 expression-based BAT yielded better accuracy than SPT and shrimp/prawn sIgE for shrimp allergy and prawn allergy (all *p* < 0.05). For discriminating shrimp allergy, the AUCs of SPT and shrimp/prawn sIgE were 0.53 (0.47–0.86, *p* = 0.19) and 0.57 (0.38–0.76, *p* = 0.49), respectively. Regarding prawn allergy, the AUCs of SPT and shrimp/prawn sIgE sIgE were 0.58 (0.39–0.76, *p* = 0.44) and 0.58 (0.41–0.75, *p* = 0.36), respectively.

Using the Youden index, the cut-off values of CD63% expression on basophils were 13.95% for shrimp and 23.90% for prawn. Among them, BAT yielded the most optimal AUC (0.88), specificity (90%), and LR+, with a cutoff of 13.95% for diagnosing allergy to shrimp. The BAT values of prawn were relatively promising, with an AUC of 0.74, and similar sensitivity/specificity values (80%/77%, 81%/70%, respectively) (Table 2).

### 3.4. Combination of BAT and SPT, Shrimp/Prawn sIgE in Discriminating Shellfish-Allergic from Non-Allergic Subjects

In Figure 2, the potential combination of the skin prick test and CD63-based BAT was evaluated. Only among shrimp-allergic subjects could the combination of SPT and CD63-based BAT enhance the diagnostic power, with AUC = 0.92 (0.82–0.99, *p* < 0.001), compared to CD63-based BAT only (AUC = 0.88) (Figure 2A). For prawn, the combination of BAT and SPT was not superior to using each test individually, although the AUCs showed significant *p*-values (Figure 2B). The combination of shrimp/prawn sIgE and CD63-based BAT slightly improved the diagnostic capacity compared to CD63-based BAT only, with AUC = 0.89 for shrimp allergy and AUC = 0.76 for prawn allergy.

### 3.5. Values of BAT and Allergic Testing in Predicting Reactivity to Shrimp or Prawn

**Figure 3:**
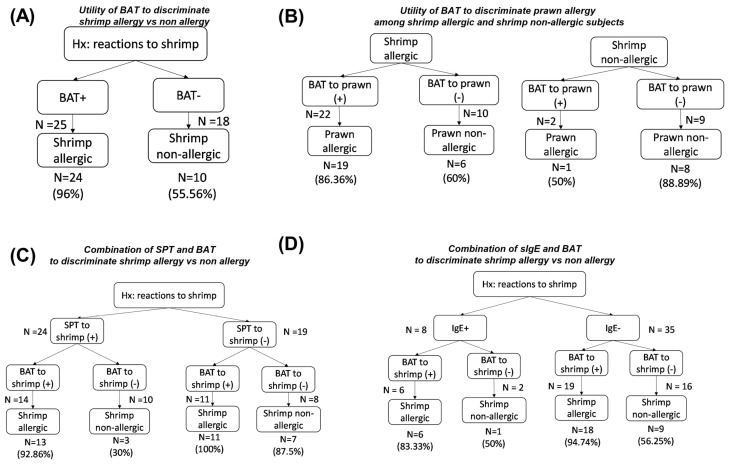
Performances of allergy tests in the diagnosis of shrimp allergy. (**A**) BAT to shrimp (cut-off 13.95%) was applied, divided into two groups: positive BAT (>13.95%) and negative BAT (≤13.95%). The proportion of challenges confirmed as shrimp-allergic and non-allergic was calculated in each positive BAT and negative BAT subgroup. (**B**) BAT to prawn (cut-off 23.9%) was used to predict the reaction to prawn in shrimp-allergic and non-allergic subjects. (**C**) The number of precise shrimp allergy diagnoses when we applied SPT first and BAT as the second diagnostic test. (**D**) Similarly, the number of precise shrimp allergy diagnoses when we applied sIgE first followed by BAT. The cut-offs for SPT and sIgE to shrimp/prawn were 3 mm and 0.35 IU/mL, respectively. BAT, basophil activation test; IgE, shrimp/prawn sIgE; SPT, skin prick test.

**Figure 4:**
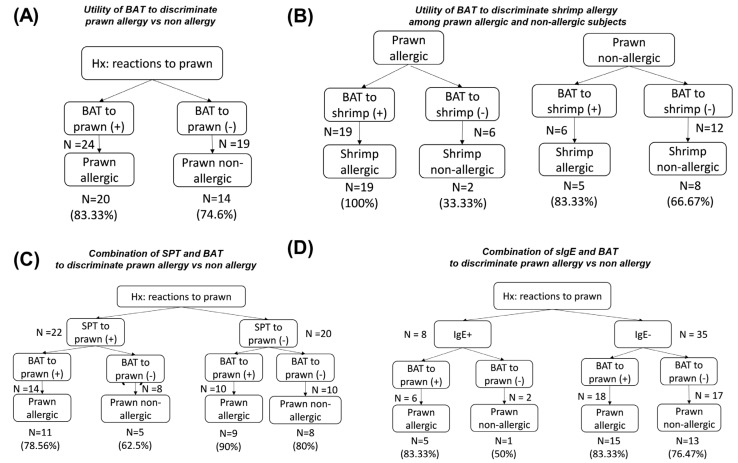
Performances of allergy tests in the diagnosis of prawn allergy. (**A**) BAT to prawn (cut-off 23.9%) was applied and divided into two groups: positive BAT (>23.90%) and negative BAT (≤23.90%). The proportion of challenges confirmed as prawn-allergic and non-allergic (Sx-) was calculated in each positive BAT and negative BAT subgroup. (**B**) BAT to shrimp (cut-off 23.9%) was used to predict the reaction to shrimp in prawn-allergic and non-allergic subjects. The number of precise prawn allergy diagnoses when we applied (**C**) SPT first or (**D**) sIgE first and BAT as the second diagnostic test. The cut-offs for SPT and sIgE to shrimp/prawn were 3 mm and 0.35 IU/mL, respectively. BAT, basophil activation test; IgE, shrimp/prawn sIgE; SPT, skin prick test.

We evaluated the diagnostic performances of SPT, sIgEs and BAT in the primary study population via two options:

- BAT as the first and only diagnostic test to diagnose shrimp or prawn allergy, as well as to predict reactions to other species;
- BAT as a second sequential step in the diagnostic process, followed by SPT or sIgE applied to shrimp/prawn as a screening test in patients with a history of IgE-mediated reactions. Figure 3 and Figure 4 depict the numbers of cases stratified by the two approaches.

#### 3.5.1. Shrimp Allergy

In Figure 3A, BAT only or BAT + SPT could precisely detect 24/32 (75%) shrimp allergies. In 19 cases with negative SPT, there were 12 cases with shrimp allergies, and 11/12 (91.67%) were discovered based on BAT (Figure 3C). While the prevalence of positive IgE to shrimp was low (8/43, 18.60%), the addition of BAT could help predict shrimp allergy in 18/19 (94.74%) cases with negative sIgE (Figure 3D).

BAT could help identify the status of prawn allergy among shrimp-allergic and non-allergic subjects. BAT was precise in either recognizing 19/22 (86.36%) co-prawn allergy among shrimp allergic subjects or in excluding 8/9 (88.89%) subjects with no prawn allergy (Figure 3C).

#### 3.5.2. Prawn Allergy

In Figure 4, regarding prawn allergy, a single BAT assay could detect 20/25 (80%) cases with allergy (Figure 4A), which is better than the combination of SPT and BAT (14/25, 56%) (Figure 4C). BAT seemed to be more valuable when using sIgE assays. Only 8/43 (18.60%) cases showed positive IgE to shrimp, and of 35 cases with negative sIgE, BAT helped precisely predict 13/17 (76.47%) true non-allergic cases with negative BAT results, and 15/18 (83.33%) true allergic cases with positive BAT results (Figure 4D). Interestingly, a positive BAT to shrimp in prawn-allergic subjects was 100% accurate in predicting shrimp allergy (Figure 4B).

## 4. Discussion

Several efforts have been made to increase the ability to accurately diagnose shellfish allergy. This study underlined BAT using in-house, local extracts for the diagnosis of a shellfish allergy in combination with conventional SPT and sIgE tests. The results are important because, in clinical practice, the available SPT and specific IgE tests using commercial extracts do not entirely match the local seafood. We proposed the optimal cut-offs for CD63-based BAT using shrimp extract (13.95%) and prawn extract (23.9%). BAT shows potential utility as the first step in predicting shrimp/prawn allergy and cross-reactivity between these two species. The use of BAT with SPT showed the best diagnostic performances for shrimp allergy.

BAT is an important in vitro test for detecting basophil responsiveness following the cross-linking of high-affinity IgE receptor-bound IgE antibodies by allergens. In this study, we have demonstrated the superior capacity of BAT in revealing shellfish allergy compared to conventional IgE immunoassays. In a recent study, BAT was found to be the most accurate test for diagnosing shrimp allergy, with higher sensitivity and specificity than conventional allergy tests, such as allergen-sIgE quantification [10]. This could be due to the fact that BAT reflects not only the level of sIgE, but also the affinity of allergens to the sIgE. Another advantage of the BAT is its ability to test an unlimited number of foods/species. For instance, a study from Japan employed BAT to identify clinical allergies in 15 fish species [28]. Similarly, in the Markers Of Nut Allergy Study (MONAS), five different nuts (peanut, cashew, hazelnut, pistachio and walnut) were tested using BAT [30]. In all the above studies, BAT showed optimal diagnostic performance ranging from 0.72–0.88 for fish allergy [28], and over 0.9 for nut allergy (0.92–0.98) [30]. The yielded AUC value of BAT for shrimp allergy in our study (0.88) is equivalent to that derived in the study on Hong Kong shrimp allergic-subjects (0.88) [10]. Tiger shrimp, giant freshwater prawn and freshwater crab are frequently consumed in Vietnam, and are common allergens among seafood-allergic subjects [21]. Therefore, the significant diagnostic performance and relatively high specificity of CD63-based BAT is clinically promising.

Several activation markers of basophils have been used in BAT to diagnose allergies in various foods. Among them, CD203c and CD63 are the most frequently used markers with different mechanisms [31]. CD203c is located on the plasma membrane and in the cytoplasmic compartments, whereas CD63 is located on the membrane of basophil granules and translocated to the cell surface after activation [31]. The activation marker that is best for the diagnosis of food allergies is controversial. Some studies have suggested a correlation between CD63 expression and the severity of allergies to peanut [32,33] and cow’s milk [34]. Other studies have demonstrated the diagnostic value of CD203c in allergies to hazelnut [35], shrimp [10] and fish [28]. Therefore, the use of activation markers depends on the adapted BAT protocol for each center. The superior role of CD63 in this study may guide the selection of an optimal BAT marker in other centers in Vietnam.

Despite many benefits, BAT assay still shows some disadvantages such as labor intensity, high cost, a small window of 24 h for sample processing after blood collection, and non-releaser basophils [36]. Therefore, conventional IgE assays, such as SPT and allergen-specific IgE assays, cannot be excluded. However, in the field of shellfish allergy, the SPT results are contradictory. Many studies have shown that SPT is a poor predictor of shellfish allergy owing to differences in shellfish extracts [17]. Only one study in Hong Kong reported that allergic subjects are an accurate predictor, with high sensitivity (>90%) but a low specificity of 28% [10]. Interestingly, we found that the combination of SPT and CD63-based BAT was better than, and improved diagnostic power compared to, using BAT or SPT alone in cases of black tiger shrimp allergy, which finding is clinically relevant.

The measurement of sIgE to tropomyosin has been reported as a useful predictor of shrimp allergy [37]. However, a study by Wai CY et al. demonstrated that BAT using shrimp extracts outperformed BAT using recombinant Pen m 1 (tropomyosin) [10]. This may be attributed to the relatively low IgE sensitization rates to shrimp tropomyosin in Asian populations—34.2% in Thailand and 37% in Japan [9,11]. Among the identified IgE-binding allergens in black tiger shrimp (*P. monodon*) and giant freshwater prawn (*M. rosenbergii*) (Table 3), both tropomyosin and arginine kinase were found in both species [38], although some studies have demonstrated the species-specific epitope of these proteins between shrimp species [39,40]. The inclusion of a diverse range of species-specific epitopes in crude extracts likely contributes to the superior diagnostic performance of BAT. Additionally, BAT showed different cutoffs for shrimp and prawn allergies. We assumed that different species-specific epitopes of allergens in each species may selectively trigger basophil degranulation.

Whether BAT can fully replace OFC in the diagnosis of shellfish allergy remains inconclusive. Among food allergies, BAT has shown the most robust diagnostic performance in peanut allergy. In particular, BAT using peanut extracts outperformed other diagnostic modalities and significantly reduced the need for OFC [52]. An in-house BAT protocol using commercial peanut extracts demonstrated 100% accuracy for the diagnosis of peanut allergy [53]. Regarding shrimp and prawn allergy, data were limited. BAT and EXiLE remained superior to SPT and sIgE for shrimp allergy [10]. Interestingly, BAT using shrimp extracts was more accurate than BAT using tropomyosin [10]. A similar trend was observed in the context of fish allergies, wherein BAT with crude fish extracts outperformed BAT using purified parvalbumin [28]. This may be attributed to the diverse array of molecular allergens present in fish and shellfish [26,41,54,55]. A positive BAT using in-house extracts may help reduce the need for OFC. However, a negative BAT result should still be confirmed with OFC. Therefore, the further optimization of BAT protocols and the standardization of shrimp and prawn allergen extracts are needed before BAT can be considered a reliable alternative to OFC in shellfish allergy diagnosis.

Several limitations should be mentioned. The sample size was relatively small, which may have affected diagnostic accuracy. A notable limitation of our study is the highly skewed distribution of total IgE levels among participants, which may have influenced the interpretation of specific IgE results to some extent. Further analysis revealed that subjects presenting with nasal symptoms or skin rash had significantly higher total IgE levels (Supplementary Materials), suggesting a potential association between elevated IgE and symptom manifestation. Recent evidence supports the use of omalizumab in the context of IgE-mediated food allergies, with dosing guided by baseline total IgE levels [56,57]. This highlights the potential relevance of omalizumab for managing shrimp or prawn allergies in Vietnamese patients, warranting further investigation. A comparison with prick–prick tests using fresh food was not included. Additionally, we did not include tropomyosin-specific IgE assays or tests of other molecular allergens due to a lack of availability. However, a previous study demonstrated that BAT to shrimp was superior to tropomyosin [10]. Lastly, the tested allergens do not represent the full spectrum of shellfish allergens in Vietnam.

## 5. Conclusions

Taken together, CD63-based BAT with crude extracts is useful for predicting clinical reactivity against shrimp and prawn. Among the diagnostic approaches, BAT alone and in combination with SPT demonstrated the highest diagnostic performance. Implementing BAT as a second-line test following SPT or sIgE enhanced overall diagnostic accuracy. While oral food challenge (OFC) remains the gold standard, BAT shows strong potential as a reliable alternative, although further optimization is still needed.

## Figures and Tables

**Table 1:** Characteristics of the recruited patients.

Characteristics	Total (N = 43)
Age	29 ± 8.66 (*)
Sex (female, %)	29 (67.44%)
Comorbid fish allergy	3 (6.98%)
Total IgE	1222 ± 3216.82 (*)
Wheal diameter of SPT to Der P (mm)	5 ± 5.10 (*)
Wheal diameter of SPT to Der F (mm)	5 ± 6.27 (*)
Shrimp allergy	32 (74.41%)
Specific allergy to shrimp	9 (26.5%)
Prawn allergy	25 (58.14%)
Specific allergy to prawn	2 (5.9%)
Both shrimp and prawn allergy	23 (67.6%)

Categorical variables are presented as N (%). (*) Continuous variables are presented as median ± SD. Der F, *Dermatophagoides farinae*; Der P, *Dermatophagoides pteronyssinus*; SPT, skin prick test.

**Table 2:** Diagnostic value of CD63 expression-based BAT used in discriminating crustacean allergy.

	Sensitivity	Specificity	Cutoff	*p*-Value	PPV	NPV	LR+	LR−
Shrimp	0.77	0.90	13.95%	<0.001 *	0.96 ± 0.04 (0.84–0.99)	0.56 ± 0.12 (0.33–0.77)	7.5	0.28
Prawn	0.88	0.77	23.9%	<0.001 *	0.83 ± 0.07 (0.66–0.95)	0.74 ± 0.1 (0.52–0.89)	3.64	0.26

The cutoffs were calculated based on the Youden’s index. *, *p*-values were calculated between allergic and non-allergic subjects using Pearson’s chi square or Fisher’s exact test. LR−, negative likelihood ratio; LR+, positive likelihood ratio; NPV, negative predictive value; PPV, positive predictive value; prawn, giant tiger prawn; shrimp, black tiger shrimp.

**Table 3:** Summary of the IgE-binding allergens identified in black tiger shrimp *P. monodon* and giant freshwater prawn *M. rosenbergii*. These allergens were reported in a previous study using similar species [38,41].

Allergens	Molecular Weight	Family	Characteristics
Myosin heavy chain	98 kDa	Class II myosins	- Encoded by *MYH* genes, with multiple isoforms expressed in tissue-specific and developmentally regulated patterns - Composed of a head (motor) domain, a neck domain, and a long coiled-coil tail - To convert chemical energy (ATP hydrolysis) into mechanical force for muscle contraction [42]
Glycogen phosphorylase	95 kDa	Glycosyl hydrolase family 65 (GH65) in the CAZy (Carbohydrate-active enzymes) classification system	- Function as a homodimer or homotetramer - To contain a pyridoxal phosphate (PLP) coenzyme (a vitamin B6 derivative) essential for catalytic activity [43]
Hemocyanin	75 kDa	Type-3 copper proteins	- Oligomeric protein composed of multiple subunits - To bind O_2_ reversibly at the two copper atoms coordinated by histidine residues - Main role in oxygen transport in open circulatory systems [44]
Enolase	50 kDa	2-phosphopyruvate hydratase	- Key enzyme involved in glycolysis - Three tissue-specific isoforms: α-enolase, which is found in most body tissues, β-enolase in muscle tissue, and γ-enolase in neuronal tissue - To catalyze the reversible dehydration of 2-phosphoglycerate (2-PG) to phosphoenolpyruvate (PEP) [45]
Aldolase	40 kDa	Aldolase	- To catalyze the reversible cleavage of fructose 1,6-bisphosphate into two three-carbon molecules - Functions as a homotetramer [46]
Arginine kinase	40 kDa	Phosphagen kinase	- To catalyze the reversible phosphorylation of arginine, forming phosphoarginine and ADP - To contain conserved domains for ATP binding and guanidino substrate (arginine) binding - To maintain ATP homeostasis during rapid energy fluctuations [47]
Tropomyosin	40 kDa	Tropomyosin	- To include actin-binding proteins that regulate actin filament function - To consist of rod-shaped dimers that polymerize along both sides of the actin filament in a head-to-tail fashion - Regulation of actin-myosin interaction in striated muscle [48]
Sarcoplasmic calcium-binding protein	22 kDa	EF-hand calcium-binding protein	- To contain one or more EF-hand motifs—helix–hoop–helix structures specialized for Ca^2+^ binding - To bind Ca^2+^ ions with high affinity and act as a calcium buffer in muscle cells - To play a key role in the relaxation of muscle by helping reduce cytoplasmic Ca^2+^ after contraction - To participate in calcium signaling and homeostasis, particularly in fast-contracting muscles such as those in the abdomen of shrimp or lobsters [49]
Reticulon-like protein	20 kDa	Reticulon	- Primarily localized to the endoplasmic reticulum (ER) - Integral membrane proteins with hairpin-like topology in the ER membrane - Involved in ER morphogenesis, particularly in generating and stabilizing curved ER tubules [50]
Fatty acid-binding protein	18 kDa	Fatty acid-binding protein	- To contain β-barrel structures, a ligand-binding site, and an N-terminal α-helix–turn–helix motif - To bind long-chain fatty acids and other hydrophobic ligands - To facilitate the intracellular transport of fatty acids to sites of metabolism (mitochondria, peroxisomes), storage (lipid droplets), signaling (nucleus or enzymes) - To regulate lipid signaling, gene expression, and energy homeostasis [51]

2-PG, 2-phosphoglycerate; ATP, adenosine triphosphate; CAZy (Carbohydrate-active enzymes); ER, endoplasmic reticulum; GH65, glycosyl hydrolase family 65; MYH, myosin heavy chain; PEP, phosphoenolpyruvate.

## Data Availability

The data presented in this study are available on request from the corresponding author due to grant restrictions and confidentiality.

## Supplementary Materials

The following supporting information can be downloaded at: https://www.mdpi.com/article/10.3390/medicina61061040/s1, Figure S1. Illustration of protein bands in crude extracts from shrimp and prawn. (A) SDS-PAGE visualized the protein bands. The proteins were expressed relative to each other in terms of molecular weight (y axis) and density (x axis) in (B) shrimp and (C) prawn extracts; Figure S2. The frequency of clinical symptoms after exposure to shrimp/prawn. Data were shown as percentage. Table S1. The association of nasal symptoms and skin rash with total IgE levels.

## Abbreviations

The following abbreviations are used in this manuscript:

BAT	Basophil activation test
IgE	Immunoglobulin E
Prawn	Giant tiger prawn
Shrimp	Black tiger shrimp
SPT	Skin prick test
sIgE	Specific IgE
