# Supplementary material for: Integrated Diagnostic Approach Using Basophil Activation Test and IgE Assays for Shrimp and Prawn Allergy
Source: Medicina (Kaunas). 2025 Jun 5;61(6):1040. doi: 10.3390/medicina61061040 (PMC12195042; doi:10.3390/medicina61061040)
Supplement: Supplementary file 1 [file medicina-61-01040-s001.zip › medicina-3605778-supplementary.docx]

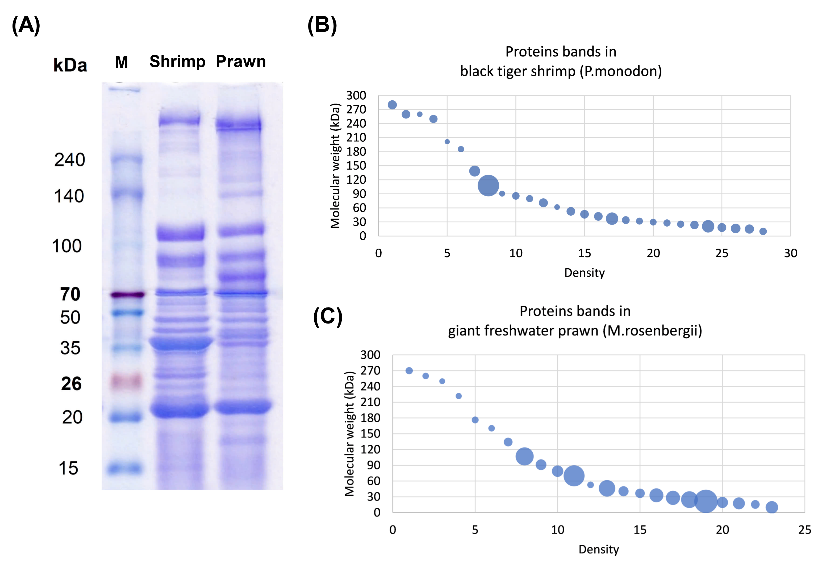


**Supplementary Figure S1. Illustration of protein bands in crude extracts from shrimp and prawn. (A) SDS-PAGE visualized the protein bands. The proteins were expressed relative to each other in terms of molecular weight (y axis) and density (x axis) in (B) shrimp and (C) prawn extracts.**

**Supplementary Figure S2. The frequency of clinical symptoms after exposure to shrimp/prawn.** Data were shown as percentage.

**Table S1. The association of nasal symptoms and skin rash with total IgE levels.**

| \|  \| **Total IgE levels (IU/mL)** \| ***P values*** \| \| --- \| --- \| --- \| \| Subjects with nasal symptoms \| 3870 ± 3457.86 \| 0.003 \| \| Without nasal symptoms \| 872.10 ± 2986.97 \| \| Subjects with skin rash \| 1417.50 ± 3486.43 \| 0.002 \| \| Without skin rash \| 84.04 ± 79.61 \| |
| --- | --- | --- | --- | --- | --- | --- | --- | --- | --- | --- | --- | --- | --- |
| Data was presented as median ± SD. *P* values were analyzed by Mann-Whitney U test |
